# Chlamydia Trachomatis Prevalence in Asymptomatic Women in Madrid: Study Findings and Their Association with Risk Factors and Mental Health

**DOI:** 10.3390/biomedicines12091999

**Published:** 2024-09-02

**Authors:** Marta Rosas Cancio-Suárez, Esther Martín-Jiménez, Mario Rodríguez-Domínguez, Ana María García Da Silva, Borja M. Fernández-Félix, Beatriz Romero-Hernández, María José Cárdenas-Isasi, Santiago Moreno, Sergio Serrano-Villar, Matilde Sánchez-Conde

**Affiliations:** 1 Infectious Diseases Department, Hospital Ramón y Cajal, Carretera de Colmenar Km 9.1, 28034 Madrid, Spain; smguillen@salud.madrid.org (S.M.); serranovillar@salud.madrid.org (S.S.-V.); 2 Ramón y Cajal Research Institute (IRYCIS), 28034 Madrid, Spain; mario@salud.madrid.org (M.R.-D.); bfernandez@salud.madrid.es (B.M.F.-F.); bromeroh@gmail.com (B.R.-H.); 3 CIBER de Enfermedades Infecciosas (CIBERINFEC), 28029 Madrid, Spain; 4 Department of Medicine, University of Alcalá de Henares, Guadalajara Campus, 28801 Alcalá de Henares, Spain; 5 Alpes Primary Care Center, 28029 Madrid, Spain; emartinjimenez@gmail.com (E.M.-J.); a.morenodasilva@gmail.com (A.M.G.D.S.); 6 Microbiology Department, Hospital Ramón y Cajal, Carretera de Colmenar Km 9.1, 28034 Madrid, Spain; mj.cardenas.isasi87@gmail.com; 7 Biostatistics Department, Hospital Ramón y Cajal, 28034 Madrid, Spain

**Keywords:** sexually transmitted infections, drug use, prevalence, risk factors, interventions, sexual health

## Abstract

Background: Chlamydia trachomatis (CT) is a sexually transmitted infection that requires early detection to prevent complications. This study aimed to evaluate the prevalence of CT among asymptomatic women in Spain and investigate the relationship between CT and risk factors associated with sexual practices, as well as factors such as stress and depression. Results: We found that 3.8% of asymptomatic women tested positive for CT. Our findings suggested that having more than five sexual partners increases the risk of sexually transmitted infections (STIs) by 3.87 times when compared with having fewer partners (p = 0.005, OR: 3.87, 95% CI 1.24–11.65). Additionally, 4.5% of participants admitted to using drugs. We found that there was a slightly higher proportion of anxiety and depression among women who tested positive for CT. Conclusions: We aimed to establish a basis for the implementation of screening in asymptomatic women. Early identification and preventive measures are crucial in minimizing the long-term complications and transmission of the disease. Sexual behavior must be recognized as a risk factor, and women’s psychological well-being should be given top priority as a vital aspect of their sexual health.

## 1. Introduction

Sexually transmitted infections (STIs) are a major global health concern and can lead to serious genital and reproductive issues. According to the World Health Organization (WHO), over one million STIs are acquired daily [1]. Chlamydia trachomatis (CT) is the most commonly reported sexually transmitted infection (STI), with almost 129 million new cases reported annually. The majority of reported CT cases occur in women under the age of 25 [1,2]. The infection can cause severe health issues in women, such as pelvic inflammatory disease (PID), ectopic pregnancy, tubal factor infertility, and chronic pelvic pain [3]. However, CT is asymptomatic in up to 90% of infections, and testing rates remain low, leading to re-infections and increased transmission [2,4]. As a result, notification data greatly underestimates the true burden of infection.

One way to address this problem is through screening. The most prevalent form of screening is opportunistic screening, performed in healthcare settings for individuals seeking medical attention. This approach has been used in the USA, Sweden, and England [5,6,7], and studies have shown a reduction in CT rates, as well as rates of PID and ectopic pregnancy over time. However, despite the implementation of opportunistic screening, CT rates in Sweden, the rest of Europe, and the U.S. have continued to increase since the mid-1990s [1,2]. This may be due to targeting a larger number of people.

Currently, screening is primarily focused on women under the age of 25, and in Europe, most countries do not perform such screening in a standardized way [7,8,9]. Additionally, the lack of prevalence data in asymptomatic women makes it challenging to support the necessity for screening. Consequently, there is a gap in our understanding of the full extent of this infection. Acquiring reliable prevalence data in women from the general population will give us a clear view of the problem’s scope and assist in formulating screening recommendations.

We aimed to fill this gap by examining the prevalence of CT in asymptomatic women in the general population in Madrid, Spain. Furthermore, by conducting a structured survey, we aimed to identify the risk factors associated with CT infection in this population, thereby contributing to the development of effective preventive measures in the future.

## 2. Materials and Methods

### 2.1. Ethics

This study conformed to the principles of the Declaration of Helsinki and Good Clinical Practice Guidelines and was approved by the Independent Ethics Committee of the coordinating center Hospital Ramón y Cajal (ceic.hrc@salud.madrid.org, project number 255/21) and the participating centers. All the participants gave informed consent before the initiation of the study procedures.

### 2.2. Study Population

Between 10 July 2022 and 10 June 2023, we systematically offered study participation to all women who went for routine cervical cytology at their Primary Care Center assigned to the project in Madrid, Spain, as well as Infectious Diseases consultations for another reason (not related to clinical symptoms suggestive of an STI). A total of 202 women were included.

Inclusion criteria for participants were (1) women aged 18–50 years, (2) attending routine cervical cytology or Infectious Diseases consultations, (3) able to provide informed consent, and (4) willing to complete the online survey.

Exclusion criteria were (1) current pregnancy, (2) recent antibiotic use (within the last four weeks), (3) symptoms or diagnosis of an active STI, (4) immunocompromised status, and (5) inability to complete the survey because of language barriers or cognitive impairment.

All the participants underwent endocervical sampling with a Dacron swab and an initial urine sample for polymerase chain reaction (PCR) testing for CT and other STIs. Participants were given a card containing a QR code for direct access to an online survey, which was anonymized and managed using REDCap electronic data capture tools v13.1.27 [10].

In addition, we recorded data about people’s sociodemographic characteristics, toxic substance consumption, sexual preferences and related behaviors, use of social apps, and an assessment of their stress and depression levels using the Hospital Anxiety and Depression Scale (HADS) [11].

### 2.3. Statistical Analysis

Statistical analysis proceeded according to a prespecified statistical analysis plan. Study data were collected and managed using REDCap electronic data capture tools v13.1.27 65 [10]. The prevalence of CT was reported as the percentage of positive tests, along with the 95% exact (Clopper–Pearson) confidence interval. The sample was described based on the test results for CT using absolute and relative frequencies for categorical variables, and either mean and standard deviation or median and interquartile range (25th and 75th percentiles), depending on the distribution of continuous variables. Normality and homoscedasticity assumptions were tested using the Shapiro–Wilk test and Levene’s test, respectively. To explore potential risk factors for CT infection, we compared baseline characteristics using the Student’s *t*-test or Mann–Whitney U test for continuous variables and Fisher’s exact test for categorical variables. Additionally, we reported descriptive results based on tests for any STI and performed logistic regression analyses to assess potential relationships with any positive STI as the dependent variable. The results were presented as odds ratios (ORs) with the corresponding 95% confidence intervals. We used Stata (StataCorp. 2019. 16.1. Statistical Software; Stata Corp LLC, College Station, TX, USA) for all analyses.

## 3. Results

We included a total of 202 participants, most (60.4%) from a Spanish background, followed by South America and the rest of Europe (28% and 7.2%, respectively).

We found that 3.96 (CI 1.73–7.65) of women without symptoms tested positive for CT, suggesting that a significant number of women can harbor this bacterium without displaying any signs. Moreover, we found some variables significantly associated with CT infection and other STIs.

When comparing potential risk factors for STIs, the highest odds ratio was observed in relation to the type of sexual partner. Women who engaged in sexual relationships with both men and women (WSMW) had a significantly increased risk of acquiring an STI, with an OR of 14.84 (95% CI 3.03–72.6, p = 0.001). However, it is important to note that the confidence interval for this association was quite broad, indicating variability and suggesting that while the association is strong, further research with a larger sample size is needed to confirm this finding with more precision.

Additionally, we found that age, drug use, having more than five sexual partners in the last five years, using condoms in less than 50% of sexual encounters, and having a previous STI were all statistically associated with an increased risk of acquiring CT, *Gardnerella*, or *Candida*. Younger age was particularly associated with a higher risk (OR: 0.93, 95% CI 0.88–0.98, p = 0.009), indicating that the younger the participant, the higher the risk of infection.

The association between drug use and STI acquisition was also notable, with those reporting drug use having significantly higher odds of infection (OR: 8.19, 95% CI 2.01–33.4, p = 0.003). The increased sample size, by including all women with previous STIs and not just those with CT, likely enhanced the statistical power, allowing us to detect these significant associations.

Finally, our study explored the relationship between mental health and STIs using the HADS. We found a slightly higher prevalence of anxiety and depression among women who tested positive for CT, although these results were not statistically significant. However, there was a significant association between asymptomatic STIs and higher depression scores (p = 0.035, OR: 1.11, 95% CI 1.01–1.22), suggesting that an individual’s mental health status may impact their vulnerability to infection. A borderline significant association was also observed between stress levels and STI status (p = 0.054, OR: 1.11, 95% CI 1.00–1.23).

Table 1 and Table 2 provide a summary of these findings.

## 4. Discussion

In our study, we found a 3.96% prevalence of CT in asymptomatic women. This prevalence is similar to or greater than previous published studies [8,12,13,14,15]. A recent meta-analysis published in BMC conducted by Huai and colleagues (13) analyzed data from 29 studies conducted worldwide between 2000 and 2019. The study examined CT prevalence in men and women across 24 worldwide countries, involving a total population of 89,886. The results revealed an overall prevalence of CT in the general population to be 2.9% (95% CI, 2.4–3.5%) with Europe having a prevalence of 2,7% (95% CI, 1.9–3.6%). In a French study (14), a sexual survey found that among the national population of sexually active individuals aged 18–24, the prevalence of CT was 3.6% (95% CI: 1.9–6.8) in women and 2.4% (95% CI: 1–5.7) in men. Meanwhile, in the United Kingdom, the second National Survey of Sexual Attitudes and Lifestyles [15] reported a CT prevalence of 3% (95% CI: 1.7–5) in women and 2.7% (95% CI: 1.2–5.8) in men of the same age group. Additional research from Spain reports a prevalence rate exceeding those previously cited. The study also included sexually active men and women aged 15–24 who visited their nearest health center for STI screening. In the stratified analysis by sex, the authors found that the prevalence of CT was 4% in women (95% CI: 2.8–6.4) and 4.3% in men (95% CI: 2.9–7.2) [12]. Nevertheless, it is important to emphasize that these data were gathered from individuals, both men and women, actively seeking testing. This suggests that a significant number of patients were experiencing symptoms at the time of diagnosis. Additionally, it should be noted that the age range of the recruited patients was notably younger than the median age of our study cohort.

Therefore, based on the data from our study showing a 3.96% (95% CI 1.73–7.65) prevalence rate among a population with a median age of 33.5 years, all being asymptomatic, we emphasize the need to provide CT screening to asymptomatic women modifying current protocols, such as the Spanish ones, in which screening is primarily recommended for populations with known classic risk factors such as having multiple partners or inconsistent use of barrier methods [16]. In this context, other already published works would support the advisability of the screening for CT infection. In fact, since 2014, the U.S. Preventive Services Task Force (USPSTF) has recommended chlamydia screening for all women aged 24 years or younger who are sexually active and women older than 25 years who are at higher risk of infection [17], considering the prevalence of CT in the United States is less than 4% (2.4% according to the meta-analysis [9]). CT infection satisfies the “key criteria” for screening [18] and should be considered for this purpose. CT is a widespread disease affecting nearly 129 million new cases annually, with an increasing incidence, that can be detected early before the onset of comorbidities (such as infertility, PID, miscarriage, chronic pelvic pain, etc.) with a simple and affordable technique, which, according to data published by Honey et al., would be cost-effective [19]. This study found that chlamydia screening is cost-effective in populations with a prevalence rate ranging from 3.1% to 10% [19].

Additionally, our investigation supports previous studies that have demonstrated a correlation between CT infection and risk factors such as having multiple sexual partners, not using condoms, and past STIs [20,21,22]. Moreover, we identified unique factors suggesting a potential relationship between mental health issues like stress and depression and vulnerability to infection [23,24,25]. Consequently, we believe that expanding STI screening beyond populations with noticeable risk factors or symptoms is essential.

In addition, 4% of the participants in our study reported the use of recreational drugs, primarily MDMA, in the context of sexual intercourse. We found that drug use was associated with an 8-fold increase in the risk of acquiring an STI (OR: 8.19, 95% CI 2.01–33.4, p = 0.003). This finding aligns with our previous research, where we found that drug use and chemsex practices were highly prevalent among a population attending an STI clinic [26]. This underscores the need for further research into the role of substance use in STI risk. As recreational drug use is on the rise, especially in conjunction with sexual activity, understanding its impact on STI prevalence is crucial. Although future studies should aim to explore this relationship in more depth to inform targeted interventions and prevention strategies, these factors could be valuable in guiding the screening of asymptomatic women.

We encountered some limitations during our research, primarily due to the relatively small size of our sample and the age range of the participants, with a median age of over 25 years. This age range is often recommended for screening as it represents the population at the highest risk of infection [17]. Despite these limitations, we identified a prevalence of 3.96% of asymptomatic women testing positive for CT, indicating that the actual prevalence of CT infection in younger age groups may be much higher than previously reported. Further research with a larger and more age-diverse sample is needed to explore this issue further and validate our findings.

Moreover, we uncovered associations between specific variables such as having multiple sexual partners, unprotected sex, or previous STI and the likelihood of having a current CT infection. With a larger sample size, we could identify more associations that could assist in developing future screening strategies.

In order to create a robust screening process for asymptomatic women, it is crucial to go beyond age, identify risk factors for STI acquisition, and detect depressive symptoms during consultations. This study offers a valuable opportunity to achieve this goal and make the screening process more effective.

## 5. Conclusions

This research underscores the significance of detecting CT in women, irrespective of age, even in the absence of symptoms in our environment. Early identification and preventive measures are crucial in minimizing the long-term complications and transmission of the disease. Sexual behavior must be recognized as a risk factor, and women’s psychological well-being should be given top priority as a vital aspect of their sexual health.

## Figures and Tables

**Table 1:** Baseline characteristics.

		Chlamydia Trachomatis		p-Value
		No	Yes	
		*N* = 194	*N* = 8	
Age (*n* = 201)		39.3 (9.3)	33.5 (7.9)	0.081
Nationality (*n* = 195)	Spain	117 (62.2%)	5 (71.4%)	0.16
	Europe	14 (7.4%)	0 (0.0%)	
	South America	54 (28.7%)	1 (14.3%)	
	North America	2 (1.1%)	1 (14.3%)	
	Asia	1 (0.5%)	0 (0.0%)	
Drug consumption (*n* = 200)	No	185 (96.4%)	6 (75.0%)	0.044
	Yes	7 (3.6%)	2 (25.0%)	
Type of drug				
Cannabis		4 (2.1%)	1 (12.5%)	
Cocaine		1 (0.5%)		
MDMA or molly		5 (2.6%)		
Others		1 (0.5%)		
Smoke (*n* = 200)	Yes	41 (21.4%)	3 (37.5%)	0.38
	No	151 (78.6%)	5 (62.5%)	
Exclusive relationship (*n* = 201)	Yes	156 (80.4%)	4 (50.0%)	0.056
	No	37 (19.1%)	4 (50.0%)	
Number of sexual partners in the last 5 years (*n* = 195)	>50	1 (0.5%)	0 (0.0%)	*
	>10	15 (8.0%)	2 (25.0%)	
	>5	23 (12.3%)	3 (37.5%)	
	≤5	148 (79.1%)	3 (37.5%)	
Type of sexual relationship (*n* = 192)	WSM	178 (95.7%)	4 (66.7%)	*
	WSW	3 (1.6%)	0 (0.0%)	
	WSMW	5 (2.7%)	2 (33.3%)	
Have you had unprotected sex (including oral sex) in the last 3 months (*n* = 168)	Always	14 (8.6%)	0 (0.0%)	0.36
	In more than half	21 (13.0%)	2 (33.3%)	
	In less than half	40 (24.7%)	2 (33.3%)	
	Never	87 (53.7%)	2 (33.3%)	
Have you ever provided sexual services for money? (*n* = 200)	No	192 (100.0%)	8 (100.0%)	
	Yes	0 (0.0%)	0 (0.0%)	
Have you ever received sex for money? (*n* = 200)	No	193 (100.0%)	7 (100.0%)	
	Yes	0 (0.0%)	0 (0.0%)	
Previous STI (*n* = 199)	Yes	13 (6.8%)	2 (25.0%)	0.11
	No	178 (93.2%)	6 (75.0%)	
Type of previous STI	Chlamydia	4 (33.0%)	1 (50.0%)	
	Gonococo	1 (8.0%)	0 (0.0%)	
	Herpes	7 (58.0%)	1 (50.0%)	
HADS depression (*n* = 197)		2 (0; 6)	3.5 (1; 5)	0.594
HADS stress: (*n* = 196)		6 (2; 9)	6 (5; 16)	0.258

Note: Values not represented correspond to non-responses from patients. If a p-value is less than 0.05, it is flagged with one star (*). WSM: women sex men; WSW: women sex women; WSMW: women sex men and women.

**Table 2:** STIs and their relationship with survey results.

		ITS		OR (95% CI)	p-Value
		No	Yes		
		*N* = 181	*N* = 21		
Age (*n* = 201)		39.7 (9.2)	34.0 (8.6)	0.93 (0.88–0.98)	**
Nationality (*n* = 195)	Spain	111 (63.4%)	11 (55.0%)	Ref.	
	Europe	11 (6.3%)	3 (15.0%)	2.75 (0.67–11.37)	0.162
	South America	50 (28.6%)	5 (25.0%)	1.01 (0.33–3.06)	0.987
	North America	2 (1.1%)	1 (5.0%)	5.05 (0.42–60.2)	0.201
	Asia	1 (0.6%)	0 (0.0%)	-	-
Drug consumption (*n* = 200)	No	174 (97.2%)	17 (81.0%)	Ref.	
	Yes	5 (2.8%)	2 (9.5%)	8.19 (2.01–33.4)	**
Type of drug					
Cannabis		3 (1.7%)	2 (9.5%)		
Cocaine		1 (0.5%)	0 (0.0%		
MDMA or molly		3 (1.7%)	3 (9.5%)		
Others		1 (0.5%)	0 (0.0%)		
Smoke (*n* = 200)	No	140 (78.2%)	16 (76.2%)	Ref.	
	Yes	39 (21.8%)	5 23.8%)	1.12 (0.39–3.25)	0.834
Exclusive relationship (*n* = 201)	No	34 (18.9%)	7 (33.3%)	Ref.	0.127
	Yes	146 (81.1%)	14 (66.7%)	0.47 (0.17–1.24)	
Number of sexual partners in the last 5 years (*n* = 195)	≤5	141 (80.6%)	10 (50.0%)	Ref.	
	>5	34 (19.4%)	10 (50.0%)	4.15 (1.60–10.76)	**
Type of sexual relationship (*n* = 192)	WSM	167 (96.5%)	15 (78.9%)	Ref.	
	WSW	3 (1.7%)	0 (0.0%)	-	-
	WSMW	3 (1.7%)	4 (21.1%)	14.84 (3.03–72.6)	**
Have you had unprotected sex (including oral sex) in the last 3 months (*n* = 168)	Never	82 (54.7%)	7 (38.9%)	Ref.	
	In less than half	38 (25.3%)	4 (22.2%)	1.23 (0.34–4.47)	0.750
	In more than half	17 (11.3%)	6 (33.3%)	4.13 (1.23–13.85)	0.021
	Always	13 (8.7%)	1 (5.6%)	0.90 (0.10–7.94)	0.925
Have you ever provided sexual services for money? (*n* = 200)	No	179 (100.0%)	21 (100.0%)		
	Yes	0 (0.0%)	0 (0.0%)		
Have you ever received sex for money? (*n* = 200)	No	180 (100.0%)	20 (100.0%)		
	Yes	0 (0.0%)	0 (0.0%)		
Previous STI (*n* = 199)	No	167 (93.8%)	17 (81.0%)	Ref.	
	Yes	11 (6.2%)	4 (19.0%)	3.57 (1.03–12.45)	**
Type of previous STI	Chlamydia	4 (40.0%)	1 (25.0%)		
	Gonococo	1 (10.0%)	0 (0.0%)		
	Herpes	5 (50.0%)	3 (75.0%)		
HADS depression (*n* = 197)		2 (0; 6)	4 (1; 11)	1.11 (1.01–1.22)	**
HADS stress: (*n* = 196)		6 (2; 9)	6.5 (3; 13)	1.11 (1.00–1.23)	0.054

If a p-value is less than 0.01, it is flagged with 2 stars (**). WSM: women sex men; WSW: women sex women; WSMW: women sex men and women.

## Data Availability

The data presented in this study are available upon request from the corresponding author.
